# A lightweight YOLO-WRCS model for peanut pod detection in seed-preserving peanut shelling robots

**DOI:** 10.3389/fpls.2026.1892758

**Published:** 2026-07-14

**Authors:** Yukun Pang, Guangxin Zhao, Liqi Qiu, Wei Huo, Zecheng Zheng, Bo Cao, Ying Zhao

**Affiliations:** School of Mechanical and Automotive Engineering, Liaocheng University, Liaocheng, China

**Keywords:** deep learning, low-damage seed-preserving peanut shelling robot, peanut, peanut seed retention, YOLOv12

## Abstract

Low-damage seed-preserving peanut shelling robots require real-time recognition of peanut pod quality and orientation to guide sorting and shelling while reducing seed damage.We proposed YOLO-WRCS, a lightweight YOLOv12n-based detector integrating RDWConv, SimAM, CARAFE, and Wise IoU. A dataset of 4,201 images covering five peanut pod classes was used for training and testing. The model was evaluated through ablation experiments, comparison with mainstream detectors, embedded deployment, and cross-variety validation.YOLO-WRCS achieved Precision, Recall, mAP50, and mAP50-95 values of 87.7%, 87.6%, 93.8%, and 92.7%, improving YOLOv12n by 2.2, 5.5, 3.4, and 3.2 percentage points. FLOPs and model size were reduced by 19.0% and 16.4%. On a Jetson Orin Nano platform, the model achieved 93.46% average recognition accuracy at 15.8 FPS and maintained above 90% accuracy across three untrained peanut varieties. YOLO-WRCS improves detection accuracy, lightweight design, and robotic deployment, although head-tail confusion and cross-variety robustness require further improvement.

## 1 Introduction

Peanuts are one of the world’s major oilseed crops and also possess significant medicinal value. With continuously growing consumer demand for peanuts, substantial quantities of peanut seeds are required to meet market needs (Barbour et al., 2017; Yang et al., 2020; Jiang et al., 2024). However, traditional peanut shelling machines cause significant damage to seeds, rendering them unsuitable for seed retention purposes (Lu et al., 2019; Prathamesh et al., 2023; Liao et al., 2024; Xu et al., 2024). Consequently, farmers in Shandong Province typically resort to manual shelling for seed preservation (Yan, 2013). Nevertheless, with the accelerating aging of China’s rural population and rising labor costs (Li, 2017), this labor-intensive traditional approach can no longer meet the demands of peanut cultivation (An, 2017). In this context, low-damage seed-preserving peanut shelling robot have emerged as an efficient alternative solution. To ensure seed quality, these robots must possess intelligent perception, precise decision-making, and high operational flexibility.

The intelligent detection of peanut seed status represents a crucial aspect of low-damage seed-preserving peanut shelling robot. This involves using deep learning methods to analyze and identify peanut pod characteristics, damage levels, and other parameters, enabling the robot to accurately process pods that meet seed retention requirements.

In recent years, deep learning technologies have demonstrated broad application prospects in the agricultural domain (Beyaz and Saripinar, 2024). implemented precise classification of sugar beet seed embryos using the YOLOv4 framework on NVIDIA Jetson platforms, providing a feasible solution for real-time field detection (Pawłowski et al., 2024). employed image processing techniques and the YOLOv8 model to analyze seed position, size, and type, achieving 90.1% IoU segmentation accuracy (Kundu et al., 2022). developed a YOLOv5-based identification system for pearl millet and corn seeds that achieved 99% recall rate by classifying seeds based on shape, color, and texture characteristics (Zhang et al., 2024). proposed Sugarcane-YOLO, a YOLOv8s-based visual detection method for sugarcane bud cutting applications, which attained 97.42% accuracy while maintaining excellent speed-accuracy balance (Wang et al., 2025). propose an automated maize thinning method using computer vision and deep learning, achieving 98.84% accuracy in field tests (Hu et al., 2020). proposes a 3D infrared imaging and CNN-based apple bruise detection method, achieving 97.67% accuracy through 3D mesh feature transformation and fusion strategies.

Recent studies on peanut seed detection have yielded significant results (Jiang et al., 2024). developed a nondestructive quality detection method combining terahertz imaging with CNN, achieving 98.7% accuracy in identifying normal, moldy, defective, dry, and germinated seeds within 2.2 seconds (Liu et al., 2024). proposed SEA-YOLOv5, an improved YOLOv5 model incorporating ShuffleNetv2, ECA attention mechanism, and alpha IoU loss, which reduced parameters to 0.47 M while maintaining 98.8% detection accuracy (Yang et al., 2024). introduced a 3D-HMD method for moldy peanut identification using hyperspectral imaging and 3D convolution with attention mechanisms, significantly improving detection efficiency with 81.63% average precision.

Notably, while these studies employ nondestructive detection methods, the physical transfer process of fragile peanut seeds onto and off detection platforms may cause new damage through collisions and friction. Although the method proposed in Reference (Deng and Han, 2019; Zhang et al., 2020; Yang et al., 2023) achieves relatively accurate identification of peanut pods, its practical effectiveness is significantly constrained by the lack of optimization for dedicated seed-retaining equipment. Existing studies have mainly focused on general peanut seed quality detection or single-defect recognition, while few have addressed the fine-grained recognition of peanut pod status and orientation required by low-damage seed-preserving shelling robots. In addition, limited attention has been paid to lightweight deployment, module-level design justification, and practical robotic validation under real shelling conditions.

To address these gaps, this study develops a lightweight YOLO-WRCS model based on YOLOv12 by integrating RDWConv, CARAFE, SimAM, and Wise IoU, and deploys it on a self-developed low-damage seed-preserving peanut shelling robot for real-time peanut pod status recognition. Therefore, we propose an improved YOLOv12-based visual detection method for identifying peanut pods containing suitable seeds, specifically designed for our previously developed low-damage seed-preserving peanut shelling robot. The main contributions of this work are as follows:

1. We developed YOLO-WRCS based on the YOLOv12 architecture, incorporating Wise IoU, RDWConv, CARAFE, and SimAM. The model demonstrated consistent detection performance in tests on the Dabaisha peanut variety, showing good adaptability in complex background conditions.
2. Through ablation and comparative experiments, we systematically evaluated each module’s contribution, validating the effectiveness of the YOLO-WRCS architecture and providing valuable references for future research.
3. We successfully deployed the improved model on the low-damage seed-preserving peanut shelling robot and verified its effectiveness and robustness for the Dabaisha peanut variety through practical experiments.

## 2 Materials and methods

### 2.1 Robotic platform

**Figure 1:**
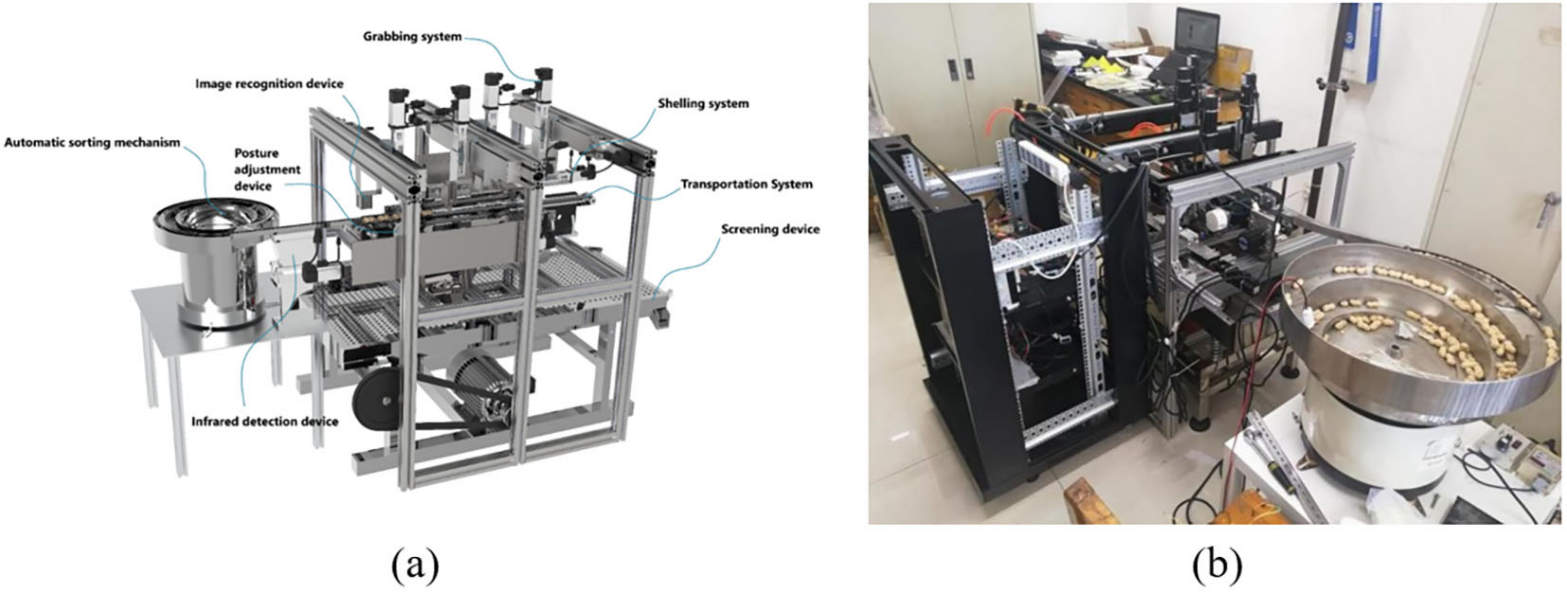
**(a)** Structural diagram of the low-damage seed-preserving peanut shelling robot. **(b)** low-damage seed-preserving peanut shelling robot.

**Figure 2:**
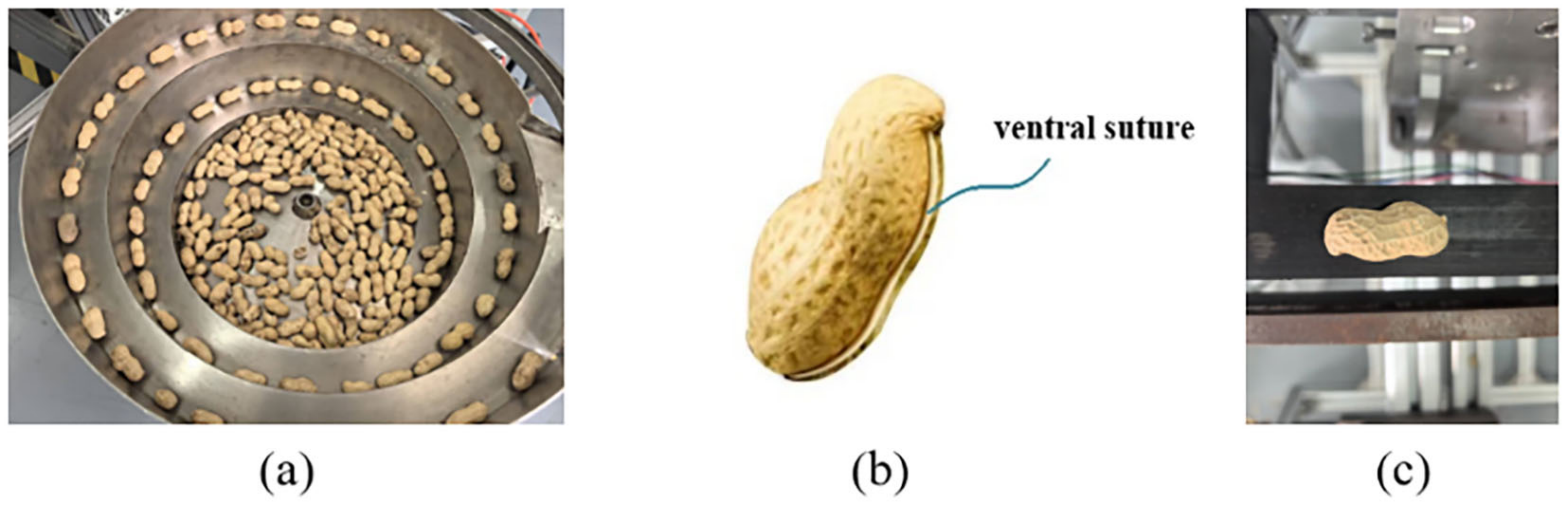
**(a)** The vibration-based sorting and feeding device. **(b)** Schematic diagram of the ventral suture of a peanut pod. **(c)** Peanut pods adjusted to the correct orientation by the vibration-based sorting and feeding device.

As shown in **Figure 1**, in our previous research (Jing, 2023), we developed a low-damage seed-preserving peanut shelling robot based on pipeline operation methodology, addressing the lack of specialized equipment in this field. The prototype’s operational process consists of six sequential stages:

1. Vibration-based sorting and feeding device: The vibration-based sorting and feeding device is designed for preprocessing peanut pods before shelling, as illustrated in **Figure 2**. Due to their irregular shape and uneven mass distribution, peanut pods exhibit an elongated form with greater mass concentrated along the ventral suture. After processing by this sorting device, the peanut pods are aligned in a stable orientation with either the head or tail facing forward and the ventral suture positioned on both sides.
2. Transportation: The conveyor system transports aligned peanuts forward continuously.
3. Visual inspection: A machine vision module performs real-time detection of peanut pods morphology and orientation to guide subsequent processing.
4. Classification & Handling: A robotic gripper separates peanut pods meeting seed criteria from the main conveyor, directing head-oriented specimens to the primary secondary conveyor and tail-oriented ones to the alternate secondary conveyor. Defective (damaged/moldy/single-seed) peanuts proceed to the terminal collection point.
5. Orientation Adjustment & Shelling: An anthropomorphic gripper mechanism was utilized for peanut pod shelling operations. Before shelling, the posture adjustment device aligns all peanuts into an upright position to prepare for the subsequent shelling process. The anthropomorphic gripper then performs a top-down shelling motion mimicking manual shelling techniques.
6. Separation: Seeds and pod fragments enter a screening system for final separation and collection.

### 2.2 Dataset acquisition

This study used the “Dabaisha” peanut variety produced in Linyi, Shandong Province, China as experimental samples. Specifically, between February and March 2025, we used a RMONCAM G200 camera mounted on the low-damage seed-preserving peanut shelling robot to randomly capture peanut images from multiple batches.

To ensure data diversity, the shooting process considers different lighting conditions and spatial distribution densities. Peanuts were delivered to the synchronous belt via a vibrating tray, and the camera on the robot’s synchronous belt was controlled to capture images. A total of 542 photos were taken during this process. To improve the model’s generalization capability, we additionally captured 325 photos under different backgrounds to supplement the dataset.

**Figure 3:**
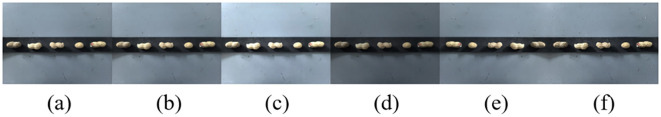
Examples of peanut pods images: **(a)** Raw image; **(b)** Resolution scaling; **(c)** Brightness enhancement; **(d)** Brightness reduction; **(e)** Image flipping; **(f)** Noise injection.

We introduced four image enhancement techniques: image flipping, resolution adjustment, brightness adjustment, and noise addition. Examples of these enhanced images are shown in the **Figure 3**. The dataset was expanded to 4,201 images and divided into training, validation, and test sets in an 8:1:1 ratio, containing 3,361, 420, and 420 images respectively.

**Figure 4:**
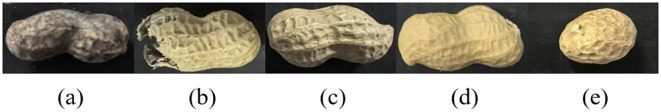
Representative peanut pod samples from the dataset: **(a)** Mold-infected; **(b)** Mechanically-damaged; **(c)** Head-oriented; **(d)** Tail-oriented; **(e)** Single-seeded.

Notably, to ensure seed quality, Shandong farmers typically exclude moldy, damaged, and single-seeded pods from seed retention. To achieve human-like shelling while minimizing seed damage, we processed tail-oriented and head-oriented peanuts separately due to their distinct mechanical properties. Based on Shandong farmers’ seed retention practices and the operational requirements of our low-damage seed-preserving peanut shelling robot, as shown in **Figure 4**, we classified the peanuts into five categories: moldy (“bad”), damaged (“break”), head-oriented (“head”), tail-oriented (“tail”), and single-seeded (“poor”). The annotation process was completed using the open-source X-AnyLabeling image annotation software. The number of each class label in the training, validation, and test sets is shown in the **Table 1**.

**Table 1:** The distribution of class labels in the dataset.

Dataset classes	Mold-infected	Mechanically-damaged	Head-oriented	Tail-oriented	Single-seeded
Training Dataset	1547	1145	6732	6426	1337
Validation Dataset	203	114	853	781	147
Test Dataset	209	113	842	800	150
Entire Dataset	1959	1372	8427	8007	1634

### 2.3 YOLOv12

As the latest object detection algorithm in the YOLO series, YOLOv12 introduces a region attention mechanism (Tian et al., 2025). Specifically, it incorporates a cross-shaped window self-attention mechanism that computes attention weights along both horizontal and vertical stripes, forming an interleaved attention structure. The region attention adopts a simple partitioning approach, dividing the feature map vertically or horizontally into L regions, which avoids complex operations while maintaining a large receptive field.

Additionally, YOLOv12 integrates a Residual Efficient Layer Aggregation Net-work (R-ELAN), whose structure is similar to Cross Stage Partial Network (CSPNet). Based on this architecture, it constructs the A2C2f module.

With these improvements, YOLOv12 surpasses all popular real-time object detectors in both accuracy and speed. The model offers five variants: n, s, m, l, and x, among which the lightweight YOLOv12n stands out as an ideal choice for resource-constrained scenarios such as low-damage seed-preserving peanut shelling robot due to its high efficiency.

### 2.4 YOLO-WRCS

Although YOLOv12 has demonstrated strong performance, we further modified its original network architecture to enhance the model’s detection accuracy for this specific task. Consequently, we propose YOLO-WRCS, an enhanced derivative of YOLOv12n, which significantly improves the recognition performance for Peanut Seed Retention Task through the following key modifications:

1. Based on DWConv, we propose RDWConv to replace partial Conv in the original model. By substituting the SiLU activation function in DWConv with ReLU6, the proposed module significantly reduces computational costs while maintaining recognition performance.
2. This study employs the Wise IoU to replace the conventional CIoU. optimizing the training process through a dynamic focusing mechanism. This allows the model to concentrate more on high-quality samples while reducing interference from occluded or boundary samples.
3. This work incorporates the SimAM adaptive attention mechanism, which achieves adaptive feature enhancement across both spatial and channel dimensions.
4. This work incorporates the CARAFE content-aware dynamic kernel generation mechanism, which better preserves edge and texture details of peanut pods during feature reorganization.

**Figure 5:**
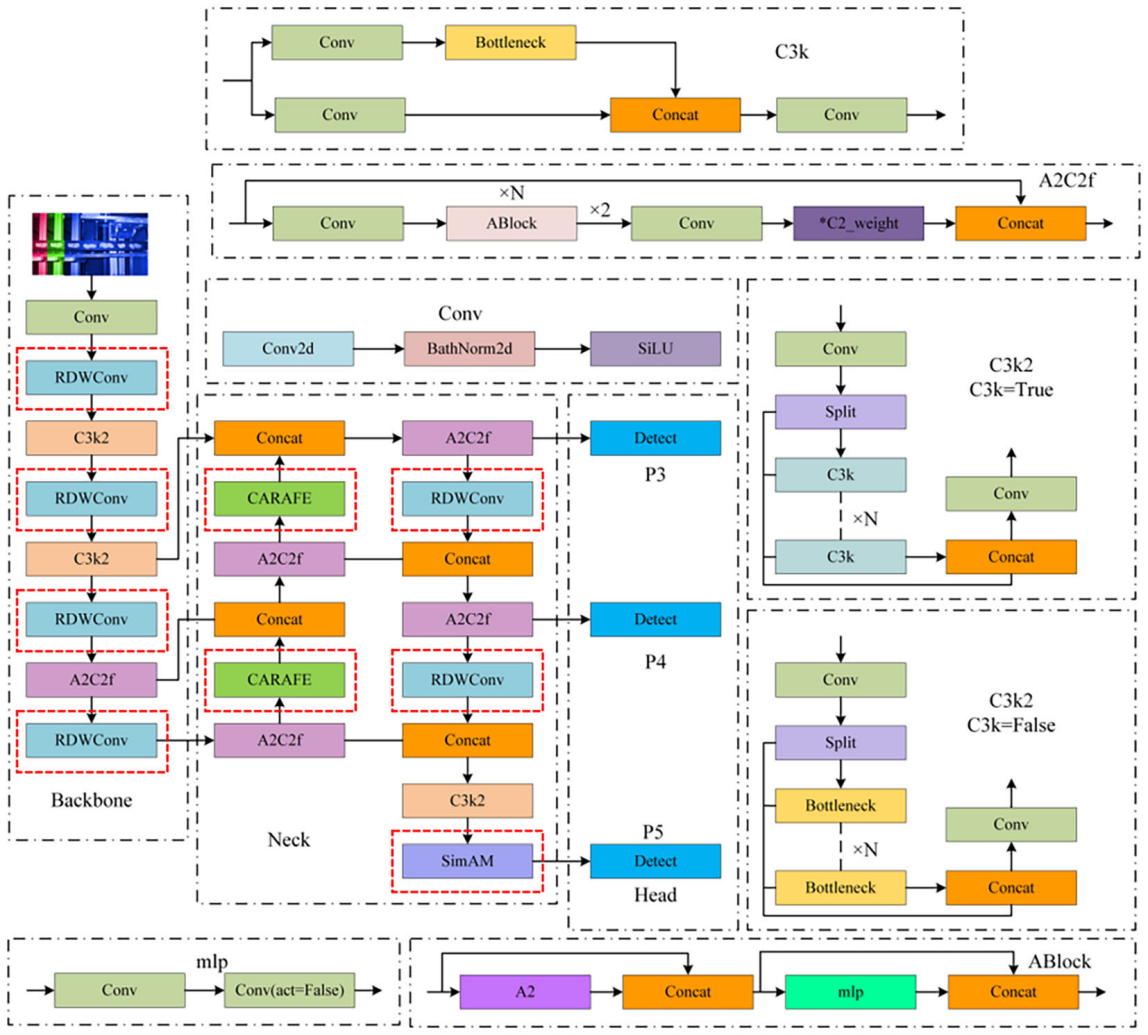
The YOLO-WRCS structural framework diagram. The red dashed box indicates the areas that have been modified compared to the original model.

The YOLO-WRCS network structure is shown in **Figure 5**.

#### 2.4.1 RDWConv

DWConv is a lightweight convolutional module designed to reduce the parameters and computational load of Conv while maintaining good feature extraction capability (Sandler et al., 2018). As one of the core components of lightweight neural networks, it decomposes standard convolution into two independent steps: depthwise convolution and pointwise convolution.

**Figure 6:**
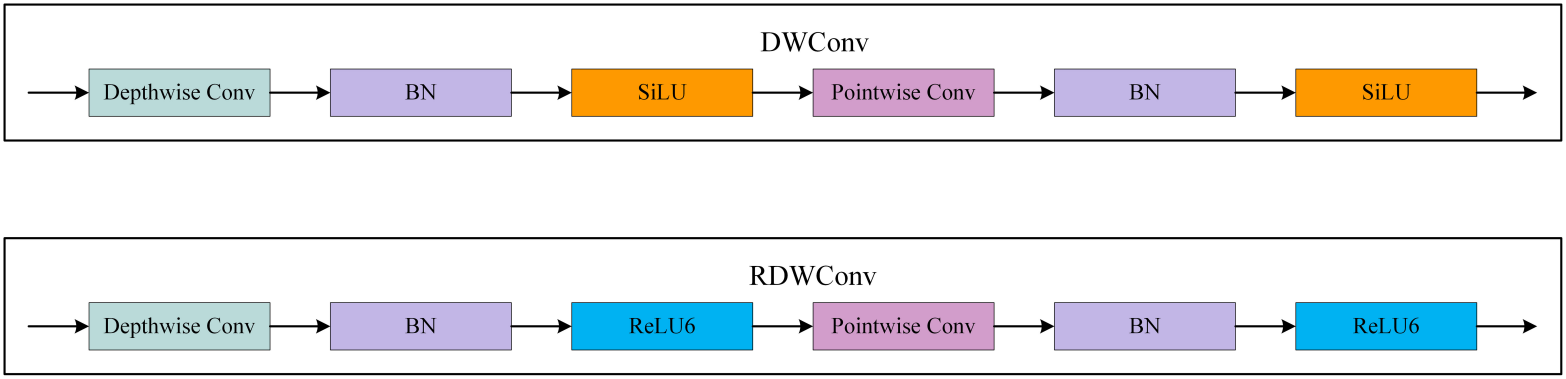
The structure of DWConv and RDWConv.

In this study, we replaced some Conv modules in the original YOLOv12 model with RDWConv, an improved version based on DWConv. The RDWConv consists of depthwise convolution, pointwise convolution, Batch Normalization (BN), and ReLU6 activation. The depthwise convolution performs individual convolution operations on each input channel, significantly reducing parameters and computations. The pointwise convolution employs 1×1 convolutions to fuse information across channels, while BN layers accelerate model convergence. The structures of DWConv and RDWConv are compared in **Figure 6**.

The SiLU possesses characteristics of being unbounded above, bounded below, smooth, and nonmonotonic. In many models, SiLU demonstrates superior performance compared to ReLU6. However, in lightweight model design, selecting activation functions requires striking a balance between computational efficiency and expressive capability.

**Figure 7:**
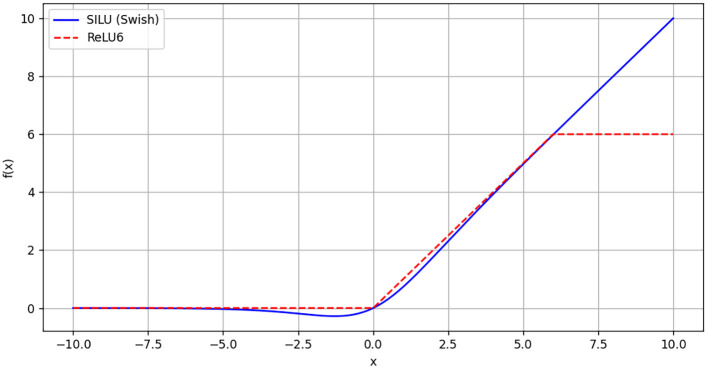
Function graphs of SiLU and ReLU6.

As shown in Equations 1, 2; **Figure 7**, ReLU6 employs simple comparison and truncation operations, resulting in lower computational overhead, making it more suitable for resource-constrained devices. In contrast, SiLU involves complex exponential operations, which impose a greater computational burden. Therefore, from the perspective of reducing computational costs and achieving model lightweighting, we chose to replace the SiLU activation function in the DWConv with ReLU6.

(1) $$\text{SiLU}(\text{x})=\text{x}\cdot \frac{1}{1+{\text{e}}^{-\text{x}}}$$

(2) ReLU6(x)=min(max(0,x),6) 

#### 2.4.2 SimAM

**Figure 8:**
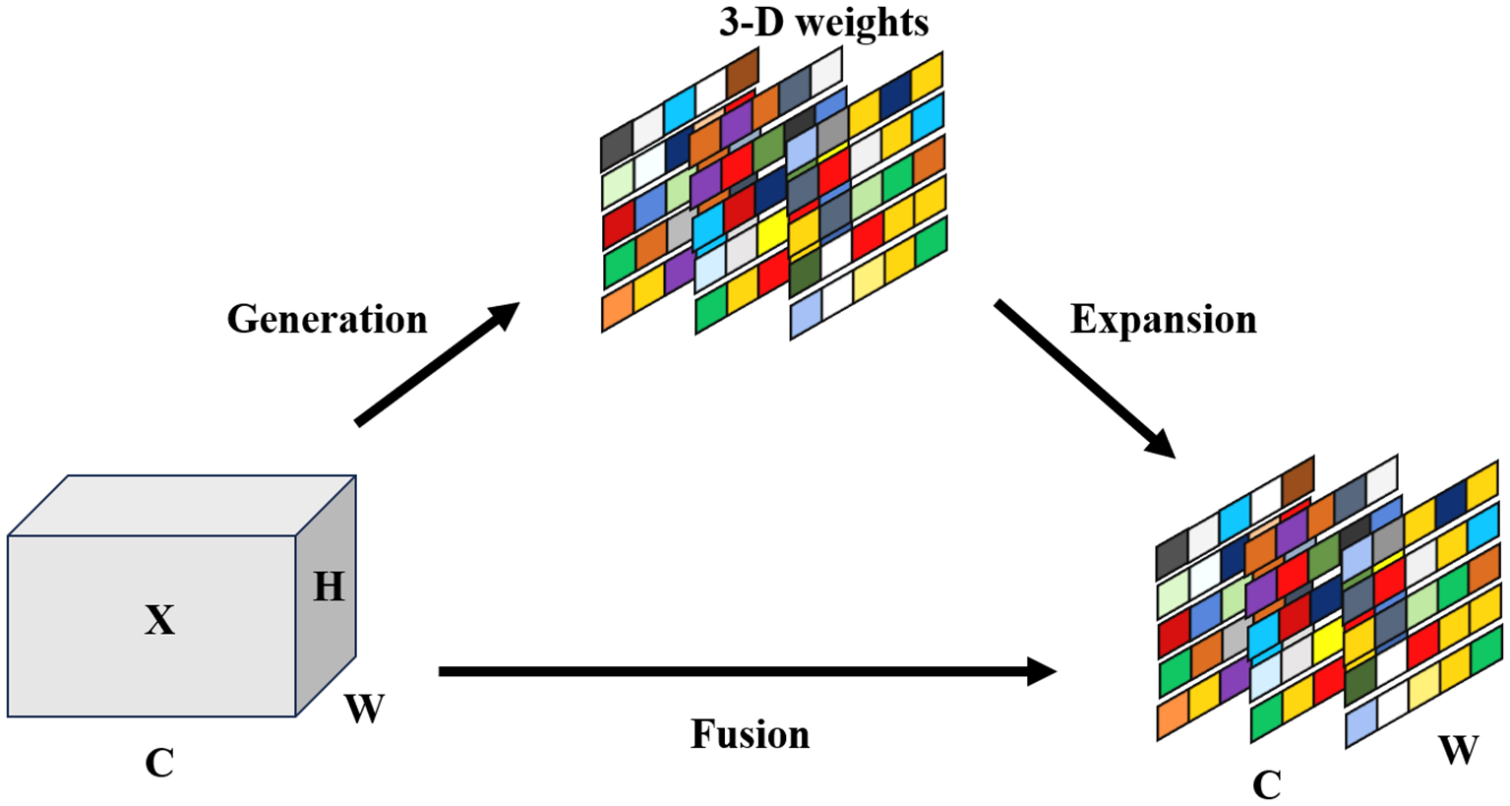
Structure diagram of SimAM.

SimAM represents a parameter-free 3D attention mechanism that effectively enhances neural networks feature extraction capabilities through computationally efficient operations (Yang et al., 2021). As illustrated in **Figure 8**, this innovative approach operates by simultaneously considering both spatial and channel dimensions to adaptively recalibrate the significance of each position within feature maps, thereby boosting model performance without introducing additional parameters. The mechanism’s core methodology involves calculating position-specific energy functions across the feature map to generate dynamic attention weights, which are then strategically applied to amplify critical features while suppressing irrelevant or noisy elements.

In the specific computational implementation of SimAM, for an input feature map $\text{X}\in {\text{R}}^{\text{B}\times \text{C}\times \text{W}\times \text{H}}$,where B represents the batch size, C denotes the number of channels, and H and W indicate the height and width of the feature map, respectively, μ and var represent the mean and variance of the feature map. These statistical measures are computed along the H and W dimensions separately. The calculation of the normalized feature map is shown in Equation 3.

(3) $$\tilde{\text{X}}=\frac{\text{X}-\text{μ}}{\sqrt{\text{var}+\text{ϵ}}}$$

Where $\tilde{\text{X}}$ represents the standardized feature map, and ϵ is a small constant added to avoid division by zero.

To quantify the similarity between pixel points, we introduce the similarity measure.

${\text{Y}}_{\text{i},\text{j}}$ as defined in Equation 4.

(4) $${\text{Y}}_{\text{i},\text{j}}=\frac{{\text{x}}_{\text{i},\text{j}}^{2}}{4(\frac{1}{\text{n}-1}\sum_{\text{k}\neq \text{i},\text{j}}{\text{x}}_{\text{k}}^{2}+\text{ϵ})}+0.5$$

Where n denotes the total number of pixels in the feature map, as mathematically defined in Equation 5.

(5) n=HW

The original feature map X is multiplied by the similarity Y to obtain the weighted feature map Z, which is then normalized to produce the feature map incorporating the attention mechanism.

In the task of peanut pod detection, especially under complex backgrounds, the edge and texture features of peanut pods are often blurred. SimAM enhances these critical features, enabling the model to locate and identify targets more accurately. At the same time, SimAM does not introduce additional computational overhead, which benefits the lightweight design of the model.

While the P3/P4 layers exhibit higher spatial resolution, applying SimAM at these levels may result in excessive attention to local noise patterns. In contrast, the P5 layer’s broader receptive field naturally focuses on target-level feature representation. We therefore strategically implement SimAM exclusively at the P5 layer to optimize detection performance.

#### 2.4.3 CARAFE

**Figure 9:**
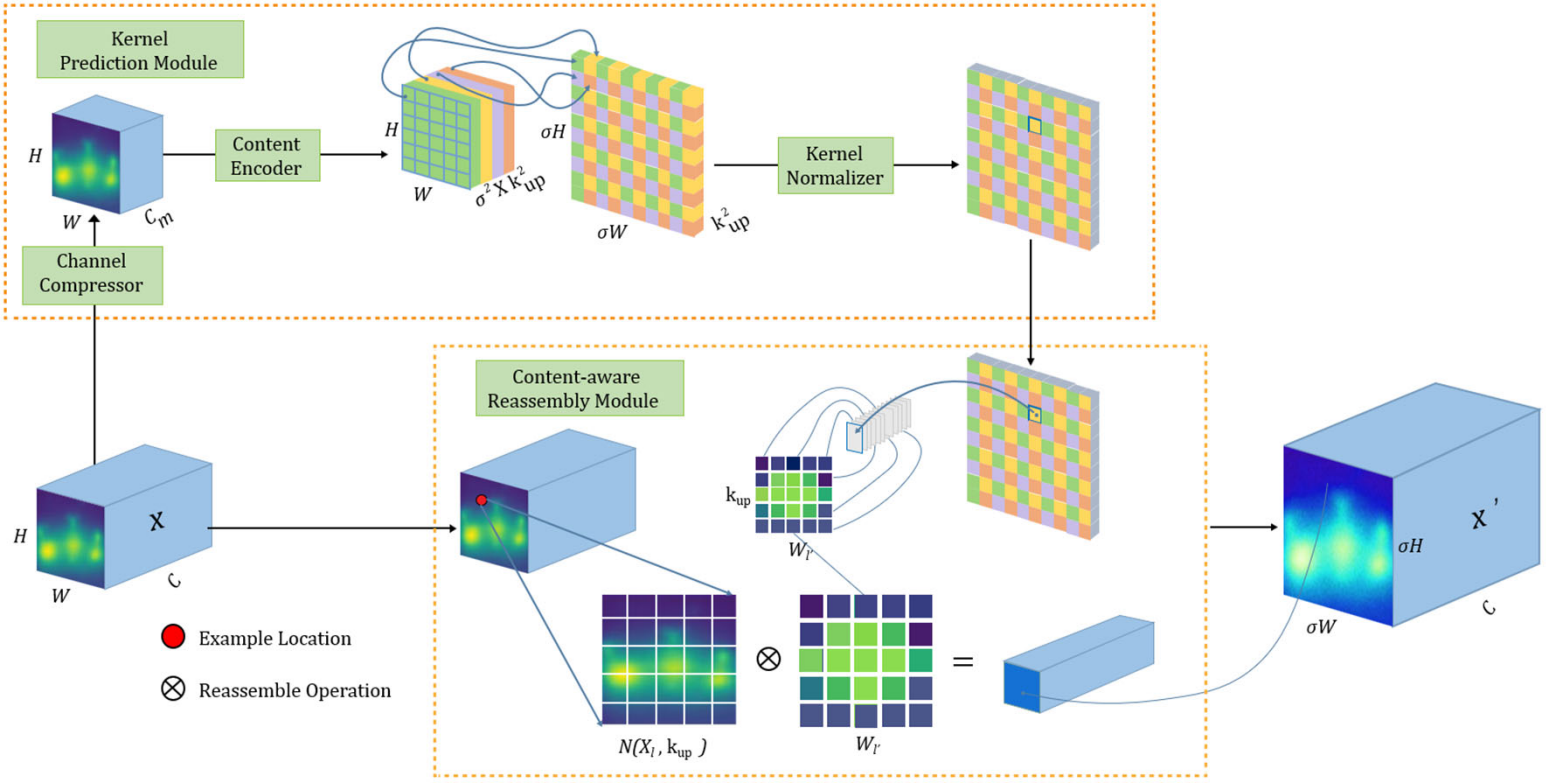
Schematic diagram of CARAFE.

In this paper, we employ CARAFE (Wang et al., 2019), an upsampling operator with a large receptive field, to replace the original upsampling module in the YOLOv12 model. As shown in **Figure 9**, in the upsampling kernel prediction module, the input feature map of size H×W×C undergoes channel compression to reduce its shape to $\text{H}\times \text{W}\times {\text{C}}_{\text{m}}$, thereby decreasing the computational cost of subsequent operations. Next, content prediction is performed to generate an upsampling kernel of size $\text{σH}\times \text{σW}\times {\text{K}}_{\text{up}}^{2}$. This kernel is then normalized to ensure the feature map’s amplitude remains unaffected, producing a weight-normalized convolution kernel. The information from the output feature map is then mapped back to the input feature map, where a ${\text{K}}_{\text{up}}\times {\text{K}}_{\text{up}}$ region centered on each position is extracted and subjected to a dot product operation with the upsampling kernel, resulting in an output feature map of size σH×σW×C. Notably, the same upsampling kernel is shared across different channels at the same spatial location. The core idea of CARAFE is to dynamically generate upsampling kernels tailored to the content of the input feature map, thereby preserving finer details during the feature upsampling process.

The main reason for selecting CARAFE is that peanut pod detection is sensitive to detailed features such as edge contours, surface textures, local damaged regions, and mold-infected areas. Traditional nearest-neighbor or bilinear upsampling methods usually rely on fixed interpolation rules, making it difficult to adaptively recover these details according to target content. In contrast, CARAFE is a content-aware upsampling method that dynamically generates reassembly kernels based on input features, enabling more effective reconstruction of pod boundaries and local abnormal regions during feature fusion.

#### 2.4.4 Wise IoU

**Figure 10:**
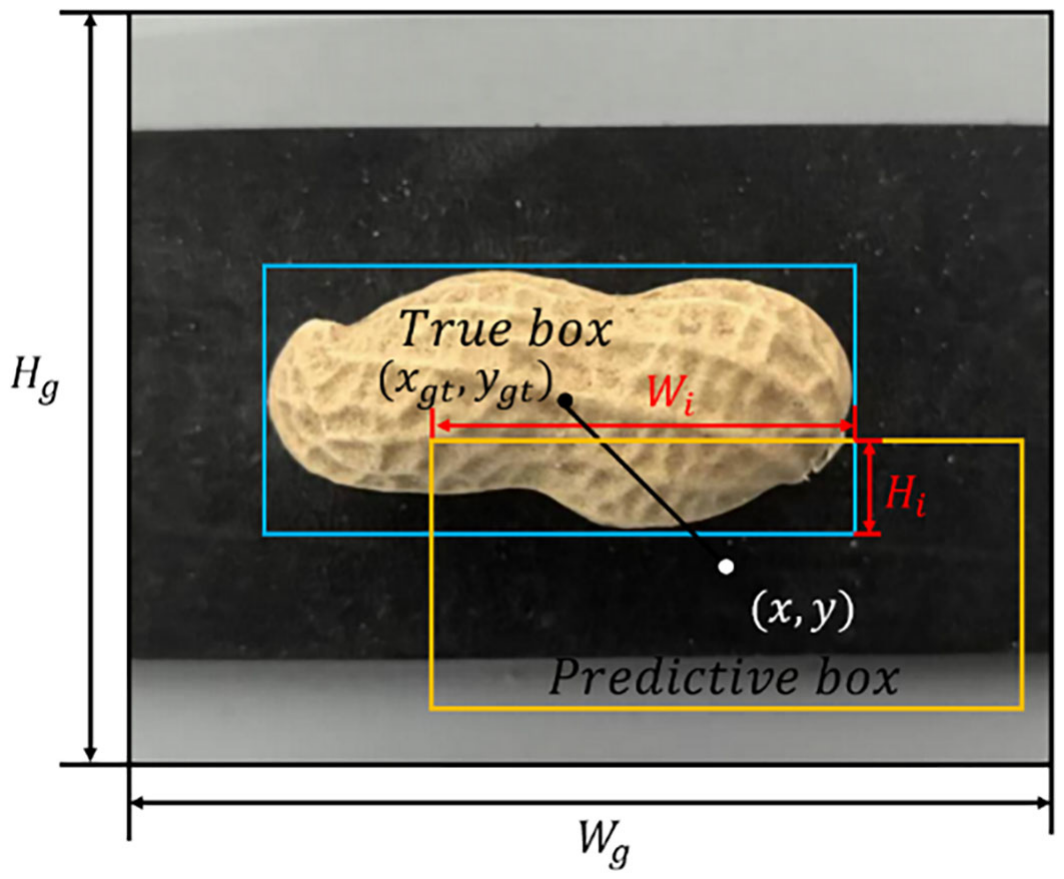
Schematic diagram of the Wise IoU loss function.

Wise IoU is a bounding box regression loss function based on a dynamic nonmonotonic focusing mechanism (Tong et al., 2023). Its core idea is to mitigate the negative impact of low-quality samples on model training by introducing a dynamic focusing mechanism, thereby improving the model’s generalization ability and detection accuracy. A schematic diagram is shown in **Figure 10**.

Compared with the CIoU, Wise IoU introduces a dynamic nonmonotonic focusing mechanism. While retaining geometric considerations such as bounding box overlap area, center-point distance, and aspect ratio, it employs an anchor quality assessment method based on outlier degree. In this study, we adopt the superior-performing WIoUv3. The Wise IoU penalty term R_WIoU_ is defined in Equation 6, the outlier degree β is calculated using Equation 7, and the WIoUv3 loss is formulated in Equation 8.

(6) $${\text{R}}_{\text{WIoU}}=\text{exp}(\frac{{\left(\text{x}-{\text{x}}_{\text{gt}}\right)}^{2}+{\left(\text{y}-{\text{y}}_{\text{gt}}\right)}^{2}}{{\left({\text{W}}_{\text{g}}^{2}+{\text{H}}_{\text{g}}^{2}\right)}^{*}})\;$$

(7) $$\text{β}=\frac{{\text{L}}_{\text{IoU}}^{*}}{\bar{{\text{L}}_{\text{IoU}}}}\in [0,+\infty )$$

(8) $${\text{L}}_{\text{WIoUv}3}={\text{rR}}_{\text{WIoU}}{\text{L}}_{\text{IoU}},\;\;\;\text{r}=\frac{\beta }{{\text{δα}}^{\text{β}-\text{δ}}}$$

Where ${\text{W}}_{\text{g}}$ and ${\text{H}}_{\text{g}}$ represent the dimensions of the minimum enclosing bounding box, $\left({\text{x}}_{\text{gt}},{\text{y}}_{\text{gt}}\right)$ and (x,y) denote the centers of the ground truth box and predicted box, respectively. ${\text{R}}_{\text{WIoU}}$ denotes the penalty term computed using Wise IoU, ${\text{L}}_{\text{IoU}}$ represents the IoU loss. β indicates the outlier degree, ${\text{L}}_{\text{IoU}}^{*}$ stands for the monotonic focusing coefficient, $\bar{{\text{L}}_{\text{IoU}}}$ represents the exponential running average with momentum m. α and δ are hyperparameters used to construct the coefficient’s nonmonotonicity. r denotes the gradient gain.

Therefore, the quality assessment criterion for anchor boxes is dynamic, enabling WIoUv3 to adaptively determine the most appropriate gradient gain allocation strategy at each timestep. This effectively suppresses the negative impact of low-quality samples on model training.

## 3 Results

### 3.1 Experimental configuration

To improve the readability and reproducibility of the experimental setup, the hardware environment, software environment, training hyperparameters, data augmentation strategies, loss coefficients, and evaluation settings were organized into structured tables. All models were trained and tested under the same experimental conditions to ensure fair comparison among different models and ablation experiments. The hardware and software environment is shown in **Table 2**, and the training and evaluation settings are presented in **Table 3**.

**Table 2:** Hardware and software environment.

Condition experimental environment	Parameters
CPU	Intel(R)Xeon(R)Platinum8474C
RAM	80 GB DDR4
GPU	GeForce RTX 4090 D
Operating system	Ubuntu 22.04.1 LTS
Languages and tools	Python 3.10, CUDA 12.6
Frameworks	PyTorch 2.6.0+cu126

**Table 3:** Training parameters.

Configuration item	Parameters
Optimizer	SGD
Initial learning rate	0.01
Batch size	64
Weight decay	0.0005
Momentum	0.937
Training epochs	200
Learning rate scheduler	Cosine annealing
Early stopping patience	50 epochs
Mosaic augmentation	Enabled, mosaic = 1
AutoAugment	randaugment
Copy-Paste augmentation	Enabled, copy_paste = 1
Random Erasing	Enabled, probability = 0.4
Box loss coefficient	7.5
Classification loss coefficient	0.5
DFL loss coefficient	1.5
Evaluation IoU threshold	0.7

### 3.2 Evaluation metrics

To evaluate the performance of the trained model, we employ the following metrics: precision (P), recall (R), mean average precision (mAP), F1-score, model size, and FLOPs. Precision and recall are calculated using Equations 9 and 10, respectively. The mAP50 and mAP50–95 metrics are defined in Equations 11 and 12, respectively, while the F1-score is calculated using Equation 13.

(9) $$\text{precision}=\frac{\text{TP}}{\text{TP}+\text{FP}}\times 100\%$$

(10) $$\text{recall}=\frac{\text{TP}}{\text{TP}+\text{FN}}\times 100\%$$

(11) $${\text{mAP}}_{50}=\frac{1}{\text{n}}\sum_{\text{i}=1}^{\text{n}}{\text{AP}}_{\text{i}}\;$$

(12) $${\text{mAP}}_{50-95}=\frac{1}{\text{m}}\sum_{\text{i}=1}^{\text{m}}\frac{1}{10}\sum_{\text{j}=1}^{10}{\text{AP}}_{\text{i},\text{i}}$$

(13) $$\text{F}1=2\cdot \frac{\text{P}\cdot \text{R}}{\text{P}+\text{R}}$$

**Figure 11:**
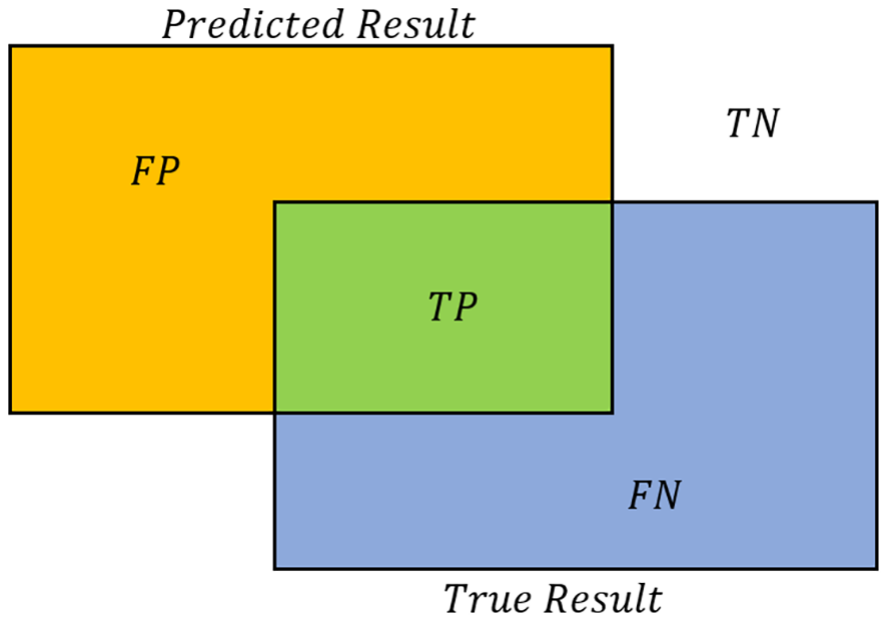
Intersection over union.

As shown in **Figure 11**, True Positives (TP) denotes cases where samples are correctly predicted as positive when they are actually positive, whereas False Positives (FP) represents cases where samples are incorrectly predicted as positive when they are in fact negative. The Average Precision for class i is defined as ${\text{AP}}_{\text{i}}$, where ${\text{AP}}_{\text{i}}$ denotes the Average Precision for class i at an IoU threshold of 0.5. ${\text{AP}}_{\text{i},\text{i}}\;$ represents the average precision for class i at an IoU threshold of 0.5+0.05×(j−1), where j is an adjustment coefficient with j∈[1,10]. The computational complexity is measured by FLOPs, where FLOPs indicates the number of floating-point operations performed by the model during operation.

### 3.3 Comparison of different loss functions

To validate the effectiveness of the loss function employed in this study (Zhang et al., 2021; Gevorgyan, 2022; Ma and Xu, 2023; Zhang and Zhang, 2023; Liu et al., 2024), we conducted comparative experiments with various loss functions after incorporating RDWConv, CARAFE, and SimAM. We evaluated the performance impacts of CIoU, SIoU, EIoU, MPDIoU, Shape IoU, Powerful IoU, and Wise IoU on the improved model. The comparative results are presented in **Table 4**.

**Table 4:** Comparison of different loss functions.

Loss functions	P%	R%	mAP50%	mAP50-95%	F1
CIoU	86.1	84.3	90.8	89.9	0.85
SIoU	89.6	82.4	90.9	90.1	0.85
EIoU	**89.9**	83.1	91.5	90.6	0.86
MPDIoU	87.1	82.5	91.5	90.7	0.84
Shape IoU	88.2	82.9	91.1	89.9	0.85
Powerful IoU	84.8	81.4	90.3	89.7	0.83
Wise IoU	87.7	**87.6**	**93.8**	**92.7**	**0.87**

Bold values indicate the best performance for each evaluation metric.

The experimental results demonstrate that while the precision achieved with Wise IoU does not show significant superiority compared to other loss functions, it exhibits remarkable advantages in several critical performance metrics, including recall, mAP50, mAP50-95, and F1 score. Comprehensive evaluation reveals that Wise IoU delivers the best performance when integrated with RDWConv, CARAFE, and SimAM in the proposed model.

It should be noted that the selection of Wise IoU was not based solely on its theoretical advantages, but also on its suitability for the peanut pod detection task. In practical shelling scenarios, peanut pods often present irregular contours, similar head-tail morphology, partial overlap, reflective surfaces, and unclear boundaries, which increase the difficulty of accurate bounding box regression. Compared with other IoU-based loss functions, Wise IoU can dynamically adjust the regression gradient according to anchor box quality, thereby reducing the negative influence of low-quality samples and improving the localization stability of medium- and high-quality samples.

Although EIoU achieved the highest precision in **Table 4**, its recall, mAP50, mAP50-95, and F1 score were lower than those of Wise IoU. For the proposed seed-preserving peanut shelling robot, recall and localization accuracy are particularly important because missed detections or inaccurate bounding boxes may affect subsequent sorting, posture adjustment, and shelling operations. Therefore, Wise IoU was finally selected as the bounding box regression loss function of YOLO-WRCS, as it provides a better balance between detection accuracy, localization robustness, and practical robotic applicability.

### 3.4 Comparative experiments between DWConv and RDWConv

Following the integration of SimAM, CARAFE, and Wise IoU, we conducted a comprehensive comparative analysis of model performance using both DWConv and RDWConv configurations, with detailed results presented in **Table 5**.

**Table 5:** Comparison of different convolutional modules.

modules	P%	R%	mAP50%	mAP50-95%	FLOPs/G	Size/MB
Conv	86.3	85.7	92.2	90.7	6.6	5.6
DWConv	85.9	84.2	92.2	91.3	5.2	**4.6**
RDWConv	**87.7**	**87.6**	**93.8**	**92.7**	**5.1**	**4.6**

Bold values indicate the best performance for each evaluation metric.

The experimental results demonstrate that compared to conventional Conv and DWConv, RDWConv benefits from its enhanced architectural design. By incorporating ReLU6 activation functions to replace the original SiLU activations, it effectively mitigates numerical instability issues while maintaining lightweight characteristics and simultaneously improving model performance.

Compared with Conv, RDWConv reduced FLOPs from 6.6 G to 5.1 G and reduced model size from 5.6 MB to 4.6 MB, corresponding to decreases of 22.7% and 17.9%, respectively. Meanwhile, RDWConv improved Precision, Recall, mAP50, and mAP50–95 by 1.4, 1.9, 1.6, and 2.0 percentage points, respectively. Compared with DWConv, RDWConv maintained the same model size of 4.6 MB and further reduced FLOPs from 5.2 G to 5.1 G, while improving Precision, Recall, mAP50, and mAP50–95 by 1.8, 3.4, 1.6, and 1.4 percentage points, respectively.

The lightweight advantage of RDWConv mainly comes from depthwise separable convolution and the use of ReLU6 instead of SiLU. Depthwise convolution reduces redundant spatial computation, while pointwise convolution preserves channel fusion capability. ReLU6 avoids the exponential operation in SiLU and further lowers computational cost. Thus, RDWConv reduces FLOPs and model size while retaining the ability to extract key peanut pod features such as contours, surface texture, damage regions, and head-tail orientation, making YOLO-WRCS more suitable for real-time deployment on the shelling robot.

These results indicate that RDWConv not only preserves the lightweight advantage of depthwise convolution, but also improves feature extraction capability. For the peanut pod recognition task, this is particularly important because different pod categories may differ only in local surface texture, edge contour, damage degree, or head-tail orientation. Therefore, RDWConv was selected as the lightweight convolutional module of YOLO-WRCS because it provides a better balance between detection accuracy, computational complexity, and model size, making the model more suitable for deployment on edge devices.

### 3.5 Effect of SimAM placement on model performance

To verify the rationality of SimAM placement, this study compared the performance of placing SimAM in the Backbone, P3, P4, and P5 after introducing CARAFE, Wise IoU, and RDWConv. The results are shown in **Table 6**.

**Table 6:** Effect of different SimAM placements on model performance.

SimAM placement	P%	R%	mAP50%	mAP50-95%	FLOPs/G	Size/MB
Backbone	86.3	84.5	90.2	89.1	5.1	4.6
P3	86.9	84.8	90.9	89.8	5.1	4.6
P4	87.5	85.5	93.1	91.4	5.1	4.6
P5	**87.7**	**87.6**	**93.8**	**92.7**	**5.1**	**4.6**

Bold values indicate the best performance for each evaluation metric.

As shown in **Table 6**, FLOPs and model size remained unchanged at 5.1 G and 4.6 MB for all placements, indicating that changing the SimAM position did not increase computational or storage cost. In terms of detection performance, placing SimAM at P5 achieved the best results, with Precision, Recall, mAP50, and mAP50–95 reaching 87.7%, 87.6%, 93.8%, and 92.7%, respectively. Compared with the Backbone placement, P5 improved these metrics by 1.4, 3.1, 3.6, and 3.6 percentage points, respectively. Compared with P4, P5 further improved Recall, mAP50, and mAP50–95 by 2.1, 0.7, and 1.3 percentage points, respectively.

Compared with P3 and P4, the P5 layer contains stronger high-level semantic information, which is more beneficial for enhancing overall pod morphology, orientation differences, and status features such as mold infection and mechanical damage. The notable improvement in Recall also indicates that SimAM at P5 can more effectively reduce missed detections. Therefore, SimAM was finally placed at the P5 layer.

### 3.6 YOLO-WRCS performance

**Figure 12:**
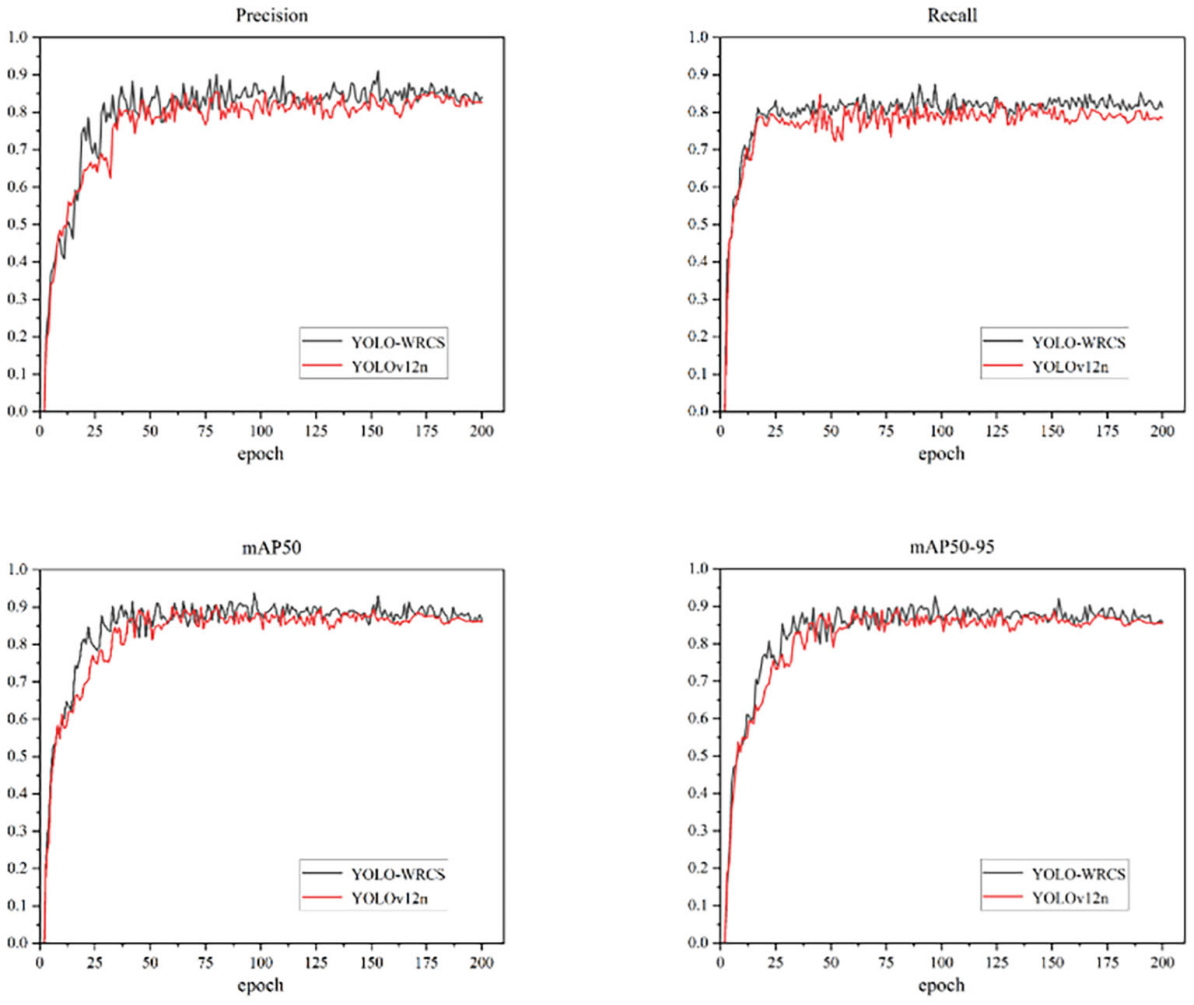
Training process curves.

As shown in **Figure 12**, the improved YOLO-WRCS model performs well in the peanut seed detection task, with a precision of 87.7%, recall of 87.6%, mAP50 of 93.8%, and mAP50–95 of 92.7%. By comparing the performance of YOLOv12n and YOLO-WRCS in terms of precision curves, recall curves, and mAP curves, it is clear that the improved model shows significant enhancements in all three key metrics. These results fully demonstrate that the YOLO-WRCS model has stronger detection capability and practical utility for peanut seed detection tasks.

Through ablation experiments, we can better understand the specific contributions of each module to the overall model. As shown in **Table 7**, “×” indicates that the module was not used, while “√” indicates that the module was employed.

**Table 7:** Ablation experiment results.

Model	W	R	C	S	P%	R%	mAP50%	mAP50-95%	FLOPs/G	Size/MB
YOLOv12n	×	×	×	×	85.5	82.1	90.4	89.5	6.3	5.5
YOLO-W	✓	×	×	×	87.2	85.6	91.9	90.7	6.3	5.5
YOLO-R	×	✓	×	×	85.2	83.7	90.2	89.2	**4.8**	**4.5**
YOLO-C	×	×	✓	×	**87.9**	84.1	92.1	90.7	6.6	5.6
YOLO-S	×	×	×	✓	86.8	84.0	92.3	91.3	6.3	5.5
YOLO-WR	✓	✓	×	×	87.6	84.7	91.4	90.1	**4.8**	**4.5**
YOLO-WRC	✓	✓	✓	×	87.4	85.9	92.3	90.7	5.1	4.6
YOLO-WRCS	✓	✓	✓	✓	87.7	**87.6**	**93.8**	**92.7**	5.1	4.6

Bold values indicate the best performance for each evaluation metric.

The experimental results demonstrate that replacing the original CIoU with Wise IoU in the YOLOv12n model led to improvements of 1.7%, 3.5%, 1.5%, and 1.2% in precision, recall, mAP50, and mAP50-95, respectively, while maintaining the same computational cost and model size, validating that Wise IoU effectively addresses sample imbalance in bounding box regression through dynamic weight adjustment. Replacing some Conv with RDWConv resulted in slight decreases of 0.3%, 0.2% and 0.3% in precision, mAP50 and mAP50-95, respectively, but reduced computational cost and model size by 1.5G FLOPs and 1.0MB, indicating that RDWConv significantly lowers model complexity with minimal impact on detection performance. Substituting the traditional upsampling module with CARAFE, despite a marginal increase in computational cost and model size, improved precision, recall, mAP50, and mAP50–95 by 2.4%, 2.0%, 1.7%, and 1.2%, respectively, proving that CARAFE better preserves feature details through dynamically generated adaptive upsampling kernels. The introduction of the SimAM attention mechanism enhanced precision, recall, mAP50, and mAP50–95 by 1.3%, 1.9%, 1.9%, and 1.8% without additional computational overhead, demonstrating its effectiveness in strengthening the model’s focus on critical features. Further experiments showed that combining Wise IoU with RDWConv improved performance while maintaining model efficiency; adding CARAFE to this configuration yielded further performance gains despite a slight increase in computational requirements; and the final YOLO-WRCS model, integrating Wise IoU, RDWConv, CARAFE, and SimAM, achieved improvements of 2.2%, 5.5%, 3.4%, and 3.2% in precision, recall, mAP50, and mAP50-95, respectively, while reducing computational cost and model size by 1.2G FLOPs and 0.9 MB compared to the original YOLOv12n, demonstrating that multimodule collaborative optimization can significantly enhance detection performance while reducing computational resource consumption.

### 3.7 Comparative analysis of different models

To comprehensively evaluate the performance of the YOLO-WRCS model, we conducted comparative experiments, with detailed results presented in **Table 8**.

**Table 8:** Comparative testing of different models.

Model	P%	R%	mAP50%	mAP50-95%	FLOPs/G	Size/MB
YOLOv5n	86.9	81.6	88.8	87.9	6.6	5.8
YOLOv8n	84.4	82.3	89.5	88.5	8.1	6.2
YOLOv9t	86.9	81.9	92.6	88.5	7.6	**4.6**
YOLOv10n	85.8	85.4	91.5	91.0	8.2	5.8
YOLO11n	85.1	86.4	92.4	91.5	6.3	5.5
YOLOv12n	85.5	82.1	90.4	89.5	6.3	5.5
SSD	86.8	84.6	87.3	86.7	269.3	95.5
RT-DETR-L	86.7	86.5	89.3	88.6	113.5	54.7
Faster R-CNN	81.4	79.8	83.6	81.4	184.9	117.1
YOLO-WRCS	**87.7**	**87.6**	**93.8**	**92.7**	**5.1**	**4.6**

Bold values indicate the best performance for each evaluation metric.

In the evaluation of peanut seed retention tasks, YOLO-WRCS shows favorable performance across multiple metrics including P, R, mAP50, mAP50-95, FLOPs, and model size, while demonstrating notable advantages particularly in FLOPs and model size. Based on the current test results obtained under specific variety and controlled conditions, comprehensive evaluation indicates that YOLO-WRCS exhibits competitive performance for this particular agricultural application scenario.

**Figure 13:**
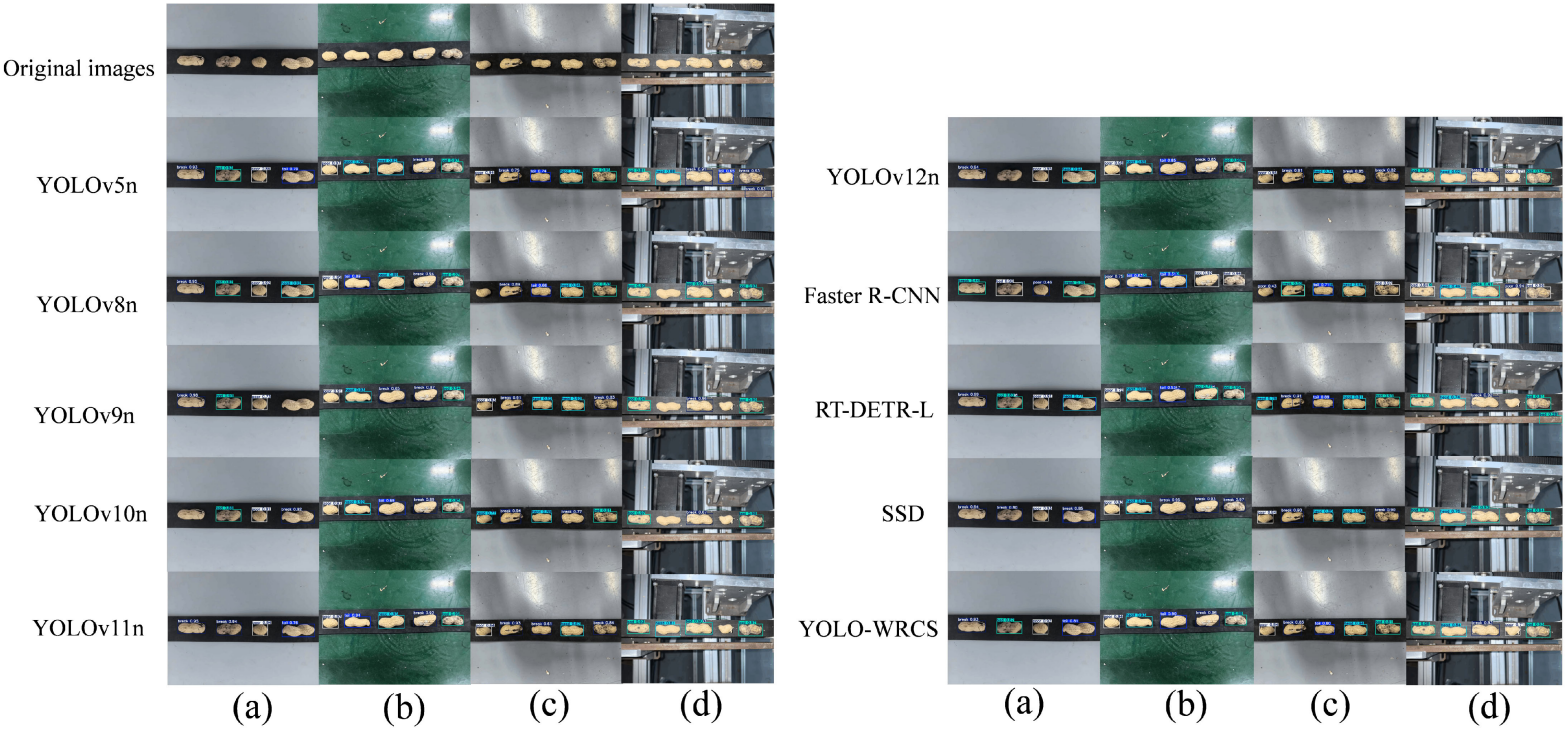
The visualization results of each model: **(a)** Under white background conditions. **(b)** Under green background with reflective interference. **(c)** Under white background with reflective interference. **(d)** Under complex background conditions.

To further validate YOLO-WRCS’s performance, we conducted visual comparison experiments against benchmark models listed in **Table 8**. The experimental results are presented in **Figure 13**.

Experimental results indicate that the YOLO-WRCS model demonstrates relatively stable peanut pod recognition performance across different testing environments. Under standard white background conditions, both YOLO-WRCS and YOLOv5n were capable of detecting peanut targets, with YOLO-WRCS showing slightly higher detection confidence than YOLOv5n. In comparison, SSD and RT-DETR-L models performed poorly in recognizing damaged and moldy peanut pods, while the Faster R-CNN model did not show significant advantages in peanut pod recognition tasks. In green background scenarios with reflective interference, YOLO-WRCS, YOLOv10n, and YOLOv12n achieved similar detection rates, with YOLO-WRCS exhibiting relatively higher confidence metrics. Under white background with reflection conditions, YOLOv5n’s detection rate was generally comparable to YOLO-WRCS, but with slightly lower confidence levels. In complex background environments, both YOLO-WRCS and YOLOv12n maintained relatively good detection rates, with YOLO-WRCS performing slightly better in terms of confidence. Comprehensive evaluation suggests that YOLO-WRCS demonstrates relatively stable recognition performance in the tests.

### 3.8 Practical deployment experiment

**Figure 14:**
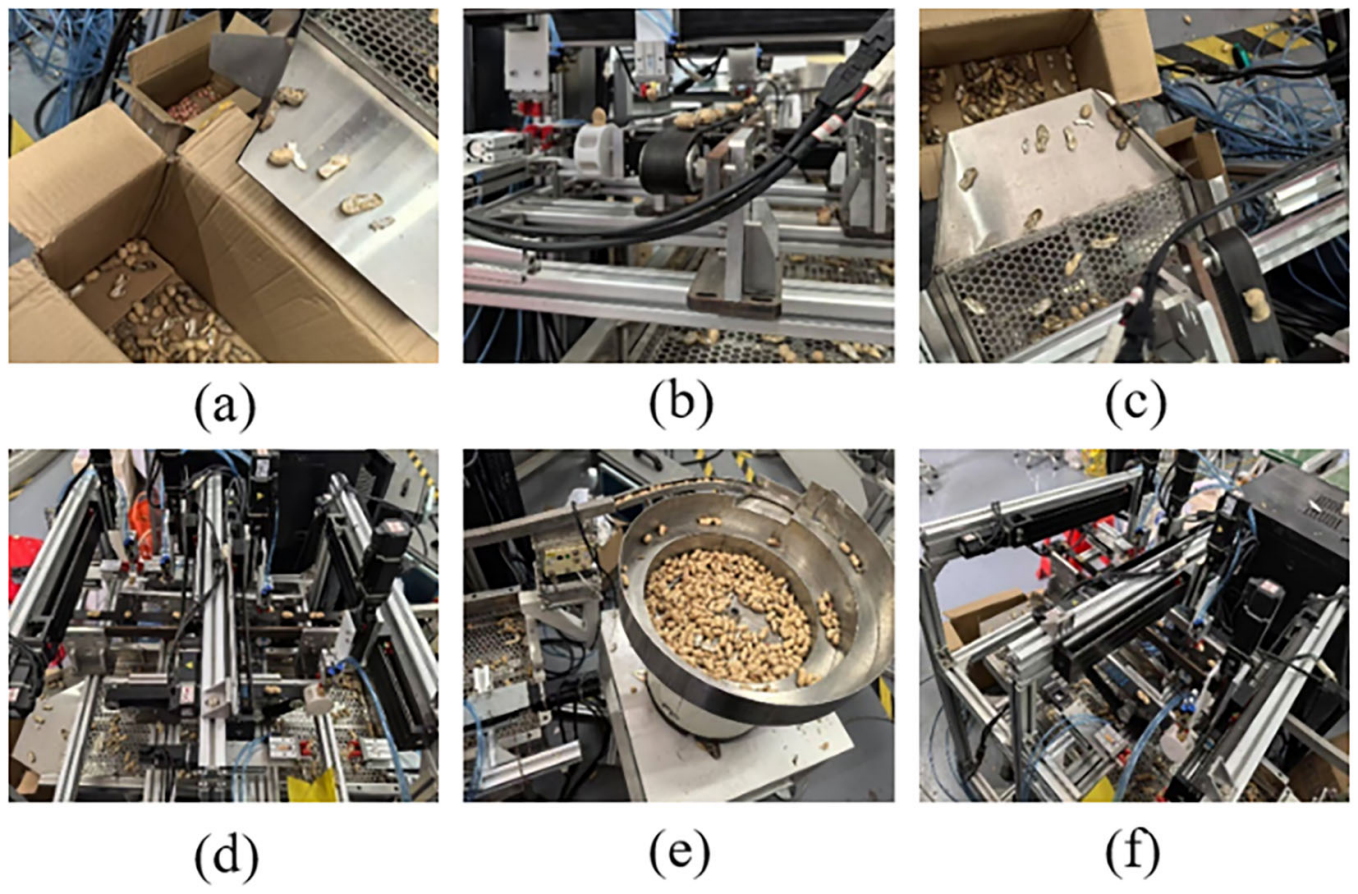
The robot equipped with YOLO-WRCS processing peanut pods.

**Figure 15:**
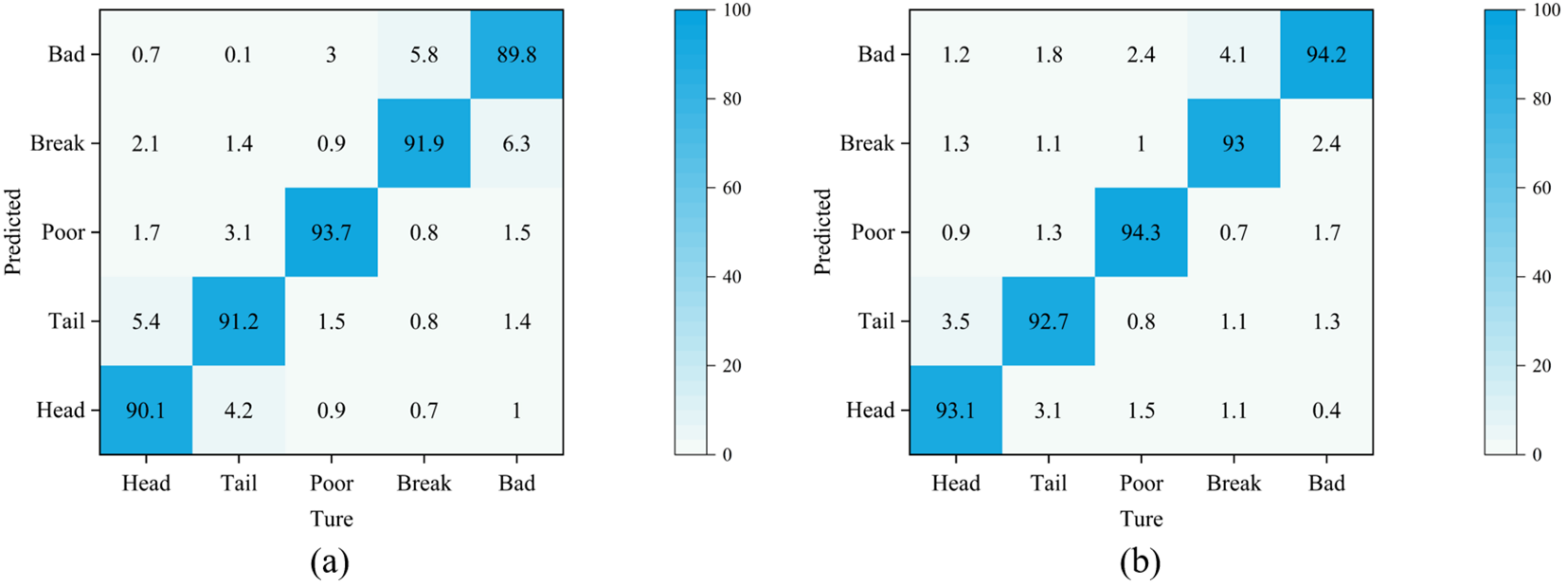
Confusion matrices from the practical deployment experiment. **(a)** Device equipped with YOLOv12n. **(b)** Device equipped with YOLO-WRCS.

To validate the effectiveness of the improved model in practical applications, as shown in **Figure 14**, we conducted field deployment experiments using our self-developed low-damage seed-preserving peanut shelling robot. A total of three separate experimental trials were conducted on February 15, 2025, September 8, 2025, and September 10, 2025, respectively, to ensure robustness and account for potential variability. For each independent trial, we prepared two test sets from the same batch of peanuts, each containing 1,500 peanut pod samples (300 samples for each pod type). The experiments focused on comparing the actual detection performance between YOLO-WRCS and the original YOLOv12n model. The experimental system used a RMONCAM G200 camera to capture the status information of peanut pods, which was then identified by the vision model to provide operational instructions for the pod processing mechanism. During normal operation, damaged and moldy samples occur at low frequencies. These sets were respectively used for the robot equipped with YOLOv12n and the robot equipped with YOLO-WRCS for comparative experiments. The samples were randomly shuffled and fed into the robotic system through the material tray. The robotic system operated based on visual recognition results. Based on the results from these three independent trials, the comprehensive performance metrics of the model are summarized in **Table 9**, and the corresponding confusion matrix is provided in **Figure 15**. This experimental design enabled a quantitative and statistically reliable evaluation of the model’s performance under real working conditions.

**Table 9:** Practical deployment experiment results.

Model	Head	Tail	Poor	Break	Bad
YOLOv12n	90.1%(± 1.3%)	91.2%(± 0.7%)	93.7%(± 0.8%)	91.9%(± 0.5%)	89.8%(± 1.2%)
YOLO-WRCS	**93.1%(± 0.5%)**	**92.7%(± 0.4%)**	**94.3%(± 0.7%)**	**93.0%(± 0.3%)**	**94.2%(± 0.7%)**

Bold values indicate the best performance for each evaluation metric.

Validation results confirm that the low-damage seed-preserving peanut shelling robot equipped with the enhanced YOLO-WRCS model demonstrates improved processing accuracy across various peanut pod types. The mean recognition accuracy for head-oriented, tail-oriented, single-seeded, damaged, and moldy peanut pods was 93.1% (± 0.5%), 92.7% (± 0.4%), 94.3% (± 0.7%), 93.0% (± 0.3%), and 94.2% (± 0.7%), respectively. These results show a measurable improvement over the original YOLOv12n model, which achieved 90.1% (± 1.3%), 91.2% (± 0.7%), 93.7% (± 0.8%), 91.9% (± 0.5%), and 89.8% (± 1.2%) for the respective pod types. Moreover, the enhanced model exhibited reduced variance across all categories, indicating more consistent performance.

The confusion matrix in **Figure 15** further shows that YOLO-WRCS improved the discrimination of different pod categories, especially for the morphologically similar head-oriented and tail-oriented pods. Compared with YOLOv12n, the misclassification rate from head-oriented to tail-oriented decreased from 5.4% to 3.5%, while that from tail-oriented to head-oriented decreased from 4.2% to 3.1%. This indicates that the improved model can reduce orientation confusion to some extent. However, such misclassification still exists and may affect subsequent sorting, posture adjustment, and shelling decisions, because an incorrect orientation judgment may cause the robot to select an inappropriate pod handling strategy. Therefore, further improvement in orientation discrimination remains necessary.

When deployed on the Jetson Orin Nano 4GB platform integrated within the robotic system, YOLO-WRCS and YOLOv12n achieved inference speeds of 15.8 FPS and 12.2 FPS, respectively. The platform is equipped with a 6-core Arm Cortex-A78AE CPU, an NVIDIA Ampere GPU with 512 CUDA cores and 16 Tensor Cores, and 4 GB 64-bit LPDDR5 memory with a bandwidth of 34 GB/s. It provides up to 20 sparse INT8 TOPS of AI performance and was operated in 10 W power mode during the experiment. The software environment was based on JetPack 6.2, including Ubuntu 22.04-based Jetson Linux, CUDA 12.6, cuDNN 9.3, and TensorRT 10.3. The input images were resized to 640 × 640 pixels during inference. YOLO-WRCS achieved an average latency of 63.3 ms/frame, while YOLOv12n achieved 82.0 ms/frame. These results indicate that the optimized model improves processing efficiency and satisfies the real-time visual recognition requirements of the robotic shelling system.

### 3.9 Model generalization experiment

To further evaluate the generalization ability of YOLO-WRCS, three peanut varieties not used in training, namely Luhua, Weihua, and Huayu, were selected as external test objects. Cross-variety practical deployment experiments were conducted on the self-developed low-damage seed-preserving peanut shelling robot platform.

The experimental setup, sample size, and statistical method were consistent with those described in Section 3.8. For each variety, three independent trials were conducted, and each trial included 1,500 peanut pod samples, with 300 samples for each category: head-oriented, tail-oriented, single-seeded, mechanically damaged, and mold-infected pods. All samples were randomly shuffled and fed into the robotic system. The results in **Table 10** are reported as the mean ± standard deviation of the three trials.

**Table 10:** Performance of YOLO-WRCS in practical deployment experiments with different peanut varieties.

Peanut variety	Head	Tail	Poor	Break	Bad
Dabaisha	**93.1%(± 0.5%)**	**92.7%(± 0.4%)**	**94.3%(± 0.7%)**	**93.0%(± 0.3%)**	**94.2%(± 0.7%)**
Luhua	91.8%(± 0.6%)	91.2%(± 0.5%)	92.9%(± 0.8%)	91.6%(± 0.6%)	93.1%(± 0.7%)
Weihua	88.7%(± 0.7%)	89.9%(± 0.6%)	91.6%(± 0.9%)	90.8%(± 1.1%)	91.9%(± 0.8%)
Huayu	90.1%(± 0.8%)	90.5%(± 0.7%)	92.0%(± 0.8%)	90.2%(± 0.8%)	92.3%(± 0.9%)

Bold values indicate the best performance for each evaluation metric.

The average recognition rate of YOLO-WRCS for the five categories was 93.46% on Dabaisha and 92.12%, 90.58%, and 91.02% on the external varieties Luhua, Weihua, and Huayu, respectively, corresponding to decreases of 1.34, 2.88, and 2.44 percentage points compared with Dabaisha. These results show that the model maintained an average recognition rate above 90% on varieties not involved in training, indicating a certain degree of cross-variety generalization ability. However, morphological differences among varieties still caused performance degradation.

At the category level, the decreases were more evident for Head and Tail. Compared with Dabaisha, the Head and Tail recognition rates decreased by 4.4 and 2.8 percentage points for Weihua, 3.0 and 2.2 percentage points for Huayu, and 1.3 and 1.5 percentage points for Luhua, respectively. This indicates that pod orientation recognition is more sensitive to varietal morphology, especially when pod-end shape, fullness, and contour proportion vary, making the model more likely to confuse Head and Tail.

In contrast, the Poor, Break, and Bad categories showed smaller fluctuations. Compared with Dabaisha, the decreases among the three external varieties ranged from 1.4 to 2.7 percentage points for Poor, 1.4 to 2.8 percentage points for Break, and 1.1 to 2.3 percentage points for Bad. This suggests that defective or abnormal pods usually have more distinct color, texture, and surface morphology features, leading to better cross-variety recognition stability.

## 4 Discussion

Through the synergistic integration of RDWConv, CARAFE, SimAM, and Wise IoU, YOLO-WRCS improves peanut pod status recognition performance while reducing computational resource consumption. Compared with the original YOLOv12n baseline, the improved model achieves a better balance among detection accuracy, lightweight design, and edge-device deployment efficiency, indicating its suitability for real-time visual recognition in low-damage seed-preserving peanut shelling robots.

However, this study still has several limitations. First, although practical deployment experiments were added for external varieties, including Luhua, Weihua, and Huayu, the training data were still mainly derived from the “Dabaisha” peanut variety, which is widely cultivated in Shandong Province. Since different peanut varieties differ in pod size, shape, surface texture, and color, the current dataset still has limited variety coverage. Therefore, the generalization ability of the model across more production areas, maturity levels, and cultivation conditions requires further validation. Future studies should continue to expand multi-variety, multi-batch, and multi-scenario datasets and conduct more systematic cross-variety and external dataset testing.

Second, misclassification still occurs between head-oriented and tail-oriented pods because of their similar morphological characteristics. This problem becomes more obvious in cross-variety testing, indicating that relying only on two-dimensional image features is still limited for pod orientation discrimination. In future work, multi-view imaging, temporal information from consecutive frames, or 3D point cloud features could be introduced to enhance the representation of pod-end morphology, contour proportion, and spatial posture, thereby reducing confusion between head-oriented and tail-oriented categories.

In addition, the robustness of the model under complex working conditions, such as extreme illumination, strong reflection, occlusion, overlapping pods, and heavily soil-adhered pod surfaces, still needs improvement. Future research could improve model stability and anti-interference capability in real shelling environments by collecting more samples under challenging conditions, optimizing data augmentation strategies, and introducing adversarial training or domain adaptation methods.

Finally, although YOLO-WRCS has achieved a lightweight design and has been validated on an embedded platform, there remains room for further optimization in terms of model parameters, inference speed, and long-term continuous operation stability. Model pruning, quantization, TensorRT acceleration, and neural architecture search could be adopted to further improve deployment efficiency on edge devices. Meanwhile, robotic validation should be extended from visual recognition accuracy to more complete end-to-end operational indicators, such as sorting accuracy, missed sorting rate, processing throughput, shelling success rate, and seed damage rate, to more comprehensively evaluate the engineering application value of the system in seed-preserving shelling scenarios.

## 5 Conclusions

This study proposed a lightweight detection model, YOLO-WRCS, based on an improved YOLOv12n architecture to meet the visual recognition requirements of a self-developed low-damage seed-preserving peanut shelling robot in complex working environments. The model includes four main improvements: RDWConv was adopted to reduce computational cost while maintaining detection accuracy; the parameter-free SimAM attention mechanism was introduced to enhance critical feature extraction; the CARAFE upsampling operator was used to optimize feature reconstruction; and the Wise IoU loss function was applied to improve bounding box regression.

Experimental results showed that, compared with the original YOLOv12n, YOLO-WRCS improved Precision, Recall, mAP50, and mAP50–95 by 2.2, 5.5, 3.4, and 3.2 percentage points, respectively, while maintaining low model complexity. In practical deployment experiments, the model achieved recognition accuracy above 90% for peanut pods with different orientations, single-seeded pods, damaged pods, and mold-infected pods, indicating that it can meet the basic requirements of low-damage seed-preserving peanut shelling robots in terms of real-time performance and recognition accuracy.

Nevertheless, this study still has some limitations. The dataset was mainly based on the “Dabaisha” variety, and cross-variety data coverage remains insufficient. Misclassification still exists between head-oriented and tail-oriented pods. The robustness of the model under complex scenarios, such as extreme illumination, heavily soil-adhered pods, and occlusion, still needs to be improved. In addition, the end-to-end operational performance of the robot requires further systematic validation. Future work will focus on expanding multi-variety and multi-scenario datasets, further optimizing the model with multi-view imaging, 3D point clouds, model pruning, quantization, and domain adaptation, and conducting more comprehensive continuous robotic operation experiments to provide more reliable visual recognition support for automated peanut seed-preserving shelling.

## Data Availability

The raw data supporting the conclusions of this article will be made available by the authors, without undue reservation.
